# Alteration of AMPA Receptor-Mediated Synaptic Transmission by Alexa Fluor 488 and 594 in Cerebellar Stellate Cells^1^,^2^,^3^

**DOI:** 10.1523/ENEURO.0109-15.2016

**Published:** 2016-06-07

**Authors:** Matthieu Maroteaux, Siqiong June Liu

**Affiliations:** Department of Cell Biology and Anatomy, LSU Health Sciences Center New Orleans, New Orleans, Louisiana 70112

**Keywords:** Alexa Fluor 488, Alexa Fluor 594, GluA2-lacking AMPA receptors, Ca-permeable AMPA receptors, cerebellar stellate cells, glutamate, synaptic transmission

## Abstract

The fluorescent dyes, Alexa Fluor 488 and 594 are commonly used to visualize dendritic structures and the localization of synapses, both of which are critical for the spatial and temporal integration of synaptic inputs. However, the effect of the dyes on synaptic transmission is not known. Here we investigated whether Alexa Fluor dyes alter the properties of synaptic currents mediated by two subtypes of AMPA receptors (AMPARs) at cerebellar stellate cell synapses. In naive mice, GluA2-lacking AMPAR-mediated synaptic currents displayed an inwardly rectifying current–voltage (*I–V*) relationship due to blockade by cytoplasmic spermine at depolarized potentials. We found that the inclusion of 100 µm Alexa Fluor dye, but not 10 µm, in the pipette solution led to a gradual increase in the amplitude of EPSCs at +40 mV and a change in the *I–V* relationship from inwardly rectifying to more linear. In mice exposed to an acute stress, AMPARs switched to GluA2-containing receptors, and 100 µm Alexa Fluor 594 did not alter the *I–V* relationship of synaptic currents. Therefore, a high concentration of Alexa Fluor dye changed the *I–V* relationship of EPSCs at GluA2-lacking AMPAR synapses.

## Significance Statement

Fluorescent dyes are commonly used to visualize dendritic structure in live neurons and to study the spatial and temporal integration of synaptic inputs. Here we report that a high concentration of the fluorescent dyes Alexa Fluor 594 and 488 altered AMPA receptor-mediated currents. Both dyes changed the current–voltage relationship of calcium-permeable AMPA receptors that lack the GluA2 subunit and reduced the EPSC amplitude. Given that the current–voltage relationship is a commonly used method to determine AMPA receptor subunit composition, the use of Alexa Fluor dyes at high concentrations is not suitable when studying AMPA receptor-mediated synaptic transmission.

## Introduction

Neurotransmitters released from presynaptic terminals are detected by postsynaptic receptors located on dendrites where synaptic information is processed. Dendritic integration of synaptic inputs depends on the electrophysiological properties and morphological characteristics of the dendrites, as well as the dendritic localization of the postsynaptic receptors (Magee, 2000; Myoga et al., 2009; Abrahamsson et al., 2012; Chadderton et al., 2014). Thus, the ability to visualize the dendritic structure is critical to our understanding of the spatial and temporal integration of synaptic inputs (Martin and Kosik, 2002; Grienberger et al., 2015). Alexa Fluor 488 (Alexa 488) and Alexa Fluor 594 (Alexa 594) are two fluorescent dyes that are commonly used to reveal the dendritic structure of neurons, from which membrane excitability and synaptic transmission are subsequently determined (Ding et al., 2009; Higley and Sabatini, 2010). This is in part due to the resistance to photo-bleaching and the high quantum yield of these molecules (Panchuk-Voloshina et al., 1999). Despite the popular use of Alexa Fluor dyes for live cell imaging and electrophysiological recordings, little is known about their effects on synaptic transmission. Therefore, this study addresses the question of whether the fluorescent dyes Alexa 488 and Alexa 594 alter excitatory synaptic transmission.

Fast excitatory synaptic transmission in the brain is mediated by AMPA receptors (AMPARs) located on dendrites and dendritic spines (Huganir and Nicoll, 2013). Of the four AMPA receptor subunits, GluA1–4, incorporation of the GluA2 subunit into an AMPAR determines a number of physiological properties (Mansour et al., 2001). AMPA receptors that lack the GluA2 subunit are permeable to calcium and can induce Ca^2+^- dependent synaptic plasticity (Gu et al., 1996; Liu and Cull-Candy, 2000; Kullmann and Lamsa, 2007; Lamsa et al., 2007). These have a larger single-channel conductance and more rapid kinetics than GluA2-containing receptors (Geiger et al., 1995; Swanson et al., 1997; Liu and Zukin, 2007). Cytoplasmic polyamines selectively block GluA2-lacking, but not GluA2-containing, receptors at depolarized potentials, rendering an inwardly rectifying current–voltage (*I–V*) relationship. Therefore, the *I–V* relationship of AMPAR-mediated currents has often been used to detect the subtype of AMPARs present at synapses (Bowie and Mayer, 1995; Donevan and Rogawski, 1995; Kamboj et al., 1995; Koh et al., 1995).

In the cerebellum, excitatory transmission at the parallel fiber–stellate cell synapse is mediated by GluA2-lacking AMPARs (Liu and Cull-Candy, 2000). Electrophysiological recordings of AMPAR-mediated currents at this synapse yields an inwardly rectifying *I–V* relationship. However, following a single exposure to acute stress, the *I–V* relationship of synaptic currents at the parallel fiber*–*stellate cell synapse changes from inwardly rectifying to nearly linear. This is due to a long-lasting increase in the expression of GluA2-containing AMPARs at this synapse (Liu et al., 2010). The ability to change the GluA2 content has allowed us to examine the effect of Alexa Fluor dyes on synaptic currents that are mediated by both GluA2-lacking and GluA2-containing receptors.

Because Alexa Fluor dyes at 10–250 µm are often used intracellularly to reveal the dendritic structure of neurons during patch-clamp recording (Sabatini et al., 2002; Brenowitz and Regehr, 2007; Albantakis and Lohmann, 2009; Mathy et al., 2009; Myoga et al., 2009; Watt et al., 2009; Zito et al., 2009; Higley and Sabatini, 2010; Smith and Häusser, 2010; Wilms and Häusser, 2015), we assessed their cytoplasmic effects on AMPAR-mediated synaptic transmission in cerebellar stellate cells. We found that the inclusion of 100 µm, but not 10 µm, Alexa Fluor dyes in the pipette solution led to a gradual increase in the amplitude of EPSCs at +40 mV, changing the *I–V* relationship from inwardly rectifying to more linear within 20 min. However, after an acute stress the synaptic GluA2 content increased and cytoplasmic Alexa 594 did not alter the *I–V* relationship of EPSCs. This indicates that intracellular Alexa Fluor dyes at a high concentration selectively altered the rectification property of synaptic currents mediated by GluA2-lacking AMPARs. We also observed a reduction in EPSC amplitude at −60 mV in cells recorded in the presence of Alexa 594 relative to controls. These results indicate that Alexa Fluor dyes can alter AMPAR-mediated synaptic transmission.

## Materials and Methods

### Slice preparation

Sagittal cerebellar slices (300 µm) were obtained from postnatal day 18 (P18) to P24 C57BL/6J male mice using a Leica VT1200 vibrating microslicer in the following slicing solution (in mm): 81.2 NaCl, 2.4 KCl, 0.5 CaCl_2_, 6.7 MgCl_2_, 1.4 NaH_2_PO_4_, 23.4 NaHCO_3_, 69.9 sucrose, and 23.3 glucose, pH 7.4, bubbled with 95% O_2_ and 5% CO_2_. Slices were kept in the following artificial CSF (ASCF; in mm): 125 NaCl, 2.5 KCl, 2 CaCl_2_, 1 MgCl_2_, 1.25 NaH_2_PO_4_, 26 NaHCO_3_, and 25 glucose, pH 7.4, at room temperature (21–23˚C) for 30–60 min before recording. All animal procedures were performed in accordance with the regulations of the LSU Health Sciences Center animal care committee.

### Electrophysiological recordings

Whole-cell patch-clamp recordings were performed using a Multiclamp 700B amplifier, a Digidata 1440A Digitizer, and pClamp 10 Software (all from Molecular Devices). Slices were viewed and fluorescence images were acquired using a Photometrics Cascade 512b Camera and µManager open source microscopy software (www.micro-manager.org; Edelstein et al., 2010).

Spontaneous EPSCs (sEPSCs) were recorded from stellate cells in cerebellar slices maintained in ASCF continuously bubbled with 95% O_2_ and 5% CO_2._ ACSF was supplemented with 100 µm picrotoxin to block GABA_A_ receptors and 5 µm 3-((*R*)-2-carboxypiperazin-4-yl)-propyl-1-phosphonic acid to block NMDA receptor-mediated currents. Pipettes (5-9 MΩ) were filled with the following cesium chloride-based solution (in mm): 130 CsCl, 2 NaCl, 1 CaCl_2_, 10 HEPES, 10 Cs-EGTA, 4 Mg^2+^-ATP, 1 Qx314, 5 TEA, and 0.1 spermine, pH 7.3. Spermine was included to identify the AMPAR subtype. Alexa Fluor dye 594 hydrazide sodium salt (catalog #A10438, Life Technologies) or Alexa Fluor dye 488 hydrazide sodium salt (catalog #A10436, Life Technologies) was dissolved in 150 mm KCl at a concentration of 10 mm and stored at −20˚C. Alexa Fluor dyes were then diluted in the pipette solution to the desired concentration (10 or 100 µm). Stellate cells were identified by their location in the outer two-thirds of the molecular layer and by the presence of spontaneous action potentials. Spontaneous EPSCs were recorded at −60 and +40 mV (for 2.5 min at each potential) at room temperature (21–23˚C) in the whole-cell voltage-clamp configuration, as well as at 0 mV to confirm the reversal potential. Recordings began immediately after obtaining the whole-cell configuration and lasted for at least 20 min. Series resistance was monitored throughout the recordings (27.9 ± 1.7 MΩ at 5 min; 32.3 ± 1.9 MΩ at 20 min; *n* = 31).

### Alexa Fluor diffusion

To estimate the diffusion of Alexa 594 (100 µm) along the dendrites of stellate cells, a series of pictures was taken at 60× magnification under fluorescent illumination (Lambda DG4, Sutter Instrument Company; excitation wavelength, 530–550 nm; emission wavelength, 590–650 nm). The images were acquired with a 1 s exposure time at a rate of approximately four images per 5 min, for 25 min (four images were taken at different focal planes in 5 min). Analysis was conducted using Fiji/ImageJ Software (http://fiji.sc; Schindelin et al., 2012). A maximum *z*-projection image of three to five stacked images was used at each time point (collected within a 5 min interval) to estimate the diffusion distance of Alexa 594 dye from the soma. Fluorescence intensity was determined by tracing the dendrite using the line scan tool and then subtracting the background (the latter was measured using the same line scan placed 2–3 µm away from the dendrite). For each time point, we examined the pixel fluorescence intensity along the dendrites, and the diffusion distance was determined as the dendritic length from the location where the fluorescence intensity rapidly decreased and returned to the baseline level.

### Fox urine exposure

Mice were placed in a chamber (33 × 23 × 15 cm) with a floor that was perforated with holes. After 3 min of habituation, a paper towel soaked in fox urine (5 ml; GreenSense) was placed underneath the chamber for 5 min. The animal was then returned to its home cage for 3 h before decapitation, and cerebellar slices were prepared for electrophysiology recordings.

### Data analysis

Analysis of sEPSCs was performed using NPM044 Software (from Stephen Traynelis, Emory University, Atlanta, GA; www.pharm.emory.edu/straynelis/Downloads/index.html). Spontaneous events were aligned by the rising phase, and the average amplitude of 10–50 events was used to calculate the rectification index (RI; RI = 1.5 × EPSC amplitude at +40 mV/amplitude at −60 mV). A difference in the frequency of sEPSCs detected at −60 mV compared with that at +40 mV could lead to an error in the calculation of the rectification index. We thus monitored the frequency of sEPSCs and found that the average frequency at 0–5 min was 0.16 ± 0.02 Hz at −60 mV, and 0.16 ± 0.03 Hz at +40 mV (*n* = 31). The frequency at 15–20 min was 0.17 ± 0.03 Hz at −60 mV, and 0.16 ± 0.03 Hz at +40 mV (see Table 3).

Statistical analysis of the group data is shown in Tables 1, 2, and 3 located before the Discussion section. Values are given as the mean ± SEM. In each figure, significance is reported as follows: *p < 0.05, **p < 0.01, and ***p < 0.001.

**Table 1:** Statistical analysis per figures

Figure	Distribution	Statistical test	Statistics	*p* Values	*N*
Figure 1					
^a^B	Normal	Paired Student’s *t* test	*t*_(8)_ = −2.587	0.032	9
^b^D	Normal	Paired *t* test	*t*_(6)_ = −4.469	0.004	7
^c^E	Normal	Paired *t* test	*t*_(6)_ = −1.889	0.108	7
^d^F	Normal	Paired *t* test	*t*_(6)_ = −2.926	0.026	7
^g^G	Normal	Paired *t* test	*t*_(6)_ = −1.555	0.171	7
^e^H	Normal	One-way repeated-measures ANOVABonferroni *post hoc* test:0-5 vs 5–10 min0-5 vs 10–15 min0-5 vs 15–20 min	*F*_(2,23)_ = 6.920 *t*_(6)_ = 1.59*t*_(6)_ = 3.646*t*_(6)_ = 3.693	0.004 0.8040.0160.008	7
^f^J	Normal	One-way repeated-measures ANOVABonferroni *post hoc* test:5 vs 10 min5 vs 15 min5 vs 20 min	*F*_(3,19)_ = 5.375 *t*_(4)_ = 1.413*t*_(4)_ = 4.115*t*_(4)_ = 4.453	0.014 0.9590.0430.025	5
Figure 2					
^h^B	Normal	Paired *t* test	*t*_(6)_ = −0.181	0.862	7
^i^C	Normal	Paired *t* test	*t*_(6)_ = −0.651	0.539	7
^j^D	Normal	Paired *t* test	*t*_(6)_ = −0.099	0.924	7
^k^F	Normal	Paired *t* test	*t*_(6)_ = −4.545	0.004	7
^l^G	Non-normal	Wilcoxon signed rank test	*z* = 1.183	0.297	7
^m^H	Normal	Paired *t* test	*t*_(6)_ = −2.957	0.025	7
Figure 3					
^n^B	Normal	Paired *t* test	*t*_(4)_ = 1.551	0.196	5
^o^C	Normal	Paired *t* test	*t*_(4)_ = −0.296	0.782	5
^p^D	Normal	Paired *t* test	*t*_(4)_ = 1.511	0.205	5
Figure 4					
^q^B	Normal	Paired *t* test	*t*_(4)_ = −0.051	0.962	5
^r^C	Normal	Paired *t* test	*t*_(4)_ = 0.741	0.500	5
^s^D	Normal	Paired *t* test	*t*_(4)_ = −1.801	0.146	5

**Table 2:** Amplitude and rectification at 0–5 min recording

Group	Distri- bution	Amplitude at −60 mV (pA)	*t* Test (vs control)	*p* Value	Amplitude at +40 mV (pA)	*t* Test (vs control)	*p* Value	RI	*N*
Control	n.a.	−50.4 ± 5.1	n.a.	n.a.	11.7 ± 0.7	n.a.	n.a.	0.37 ± 0.04	7
^t^A594 (100 µm)	Normal	−36.8 ± 3.6	*t*_(12)_ = −2.192	0.049	9.2 ± 1.1	*t*_(12)_ = 1.928	0.078	0.38 ± 0.03	7
^u^A488 (100 µm)	Normal	−39.4 ± 3.1	*t*_(12)_ = −1.871	0.086	9.8 ± 0.4	*t*_(12)_ = 2.347	0.037	0.38 ± 0.02	7
^v^A594 (10 µm)	Normal	−39.6 ± 3.8	*t*_(10)_ = −1.586	0.144	8.4 ± 0.7	*t*_(10)_ = 3.188	0.01	0.33 ± 0.03	5

n.a., Not applicable.

**Table 3:** Spontaneous EPSCs frequency per group

Group	Time 0–5 min		*p* Value (Paired *t* test)	Time 15–20 min		*p* Value (Paired *t* test)	*N*
	Frequency at −60 mV (Hz)	Frequency at +40 mV (Hz)		Frequency at −60 mV (Hz)	Frequency at +40 mV (Hz)		
Control	0.14 ± 0.02	0.12 ± 0.03	0.36	0.21 ± 0.05	0.22 ± 0.12	0.94	7
A594 (100 µm)	0.12 ± 0.03	0.10 ± 0.02	0.12	0.12 ± 0.02	0.08 ± 0.01	0.10	7
A488 (100 µm)	0.23 ± 0.10	0.27 ± 0.11	0.06	0.25 ± 0.1	0.22 ± 0.1	0.04	7
A594 +FxU	0.15 ± 0.03	0.17 ± 0.06	0.42	0.14 ± 0.02	0.12 ± 0.04	0.73	5
A594 (10 µm)	0.18 ± 0.02	0.15 ± 0.01	0.13	0.14 ± 0.02	0.13 ± 0.02	0.58	5

## Results

### *I–V* relationship of EPSCs becomes more linear during cytoplasmic application of Alexa Fluor dyes

To determine the effects of cytoplasmic Alexa Fluor dyes on glutamatergic synaptic currents, we included Alexa 594 (100 µm) in the pipette solution and recorded sEPSCs. We reasoned that Alexa Fluor dyes were likely to be present in the soma and proximal dendrites immediately after achieving the whole-cell configuration and would take time to diffuse to the distal dendrites. We started recording sEPSCs immediately after obtaining the whole-cell configuration at +40 and −60 mV, and recordings lasted for 20 min. The RI of synaptic currents was calculated from the ratio of the EPSC amplitude (1.5 × EPSC ampl_+40 mV_/EPSC ampl_−60 mV_).

**Figure 1.**
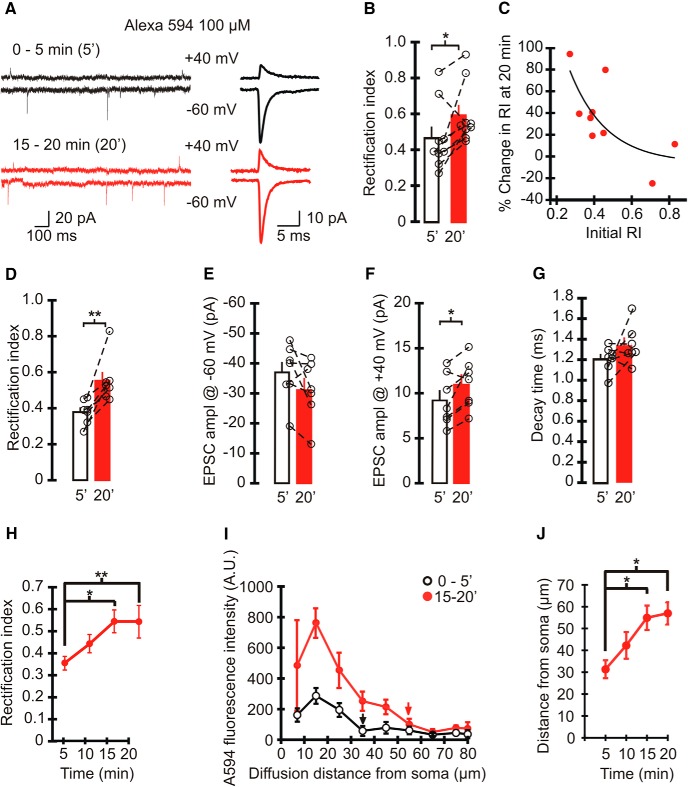
Intracellular Alexa 594 altered the rectification index of spontaneous EPSCs mediated by AMPARs in cerebellar stellate cells. ***A***, Left, Example traces of spontaneous EPSCs recorded at +40 mV (top) and −60 mV (bottom) when 100 µm Alexa 594 was included in the recording electrode. EPSC traces recorded immediately after obtaining the whole configuration (0–5 min) are shown in black, and those recorded at 15–20 min are shown in red. Right, Averaged spontaneous EPSCs. ***B***, RI of sEPSCs recorded with Alexa Fluor 594 in the patch pipette at 0–5 and 15–20 min. Average, bar graphs; individual cells, open circles. Paired *t* test, *p* < 0.05 (*n* = 9 cells, 7 animals). ***C***, Scatter plot of the percentage change in RI at 15–20 min (100 × (RI_15–20 min_ − RI_0–5 min_)/RI_0–5 min_) vs the initial RI at 0–5 min of cells filled with Alexa Fluor 594. Stellate cells with a lower initial rectification index have a greater increase in the magnitude of RI. ***D–G***, RI of sEPSC amplitudes recorded at −60 and +40 mV from cells with an initial RI < 0.5 (*n* = 7). ***D***, RI of EPSCs increased at 15–20 min of recording. Paired *t* test, *p* < 0.004. ***E***, The amplitude of sEPSCs at −60 mV did not change. ***F***, sEPSC amplitude at +40 mV increased at 15–20 min. Paired *t* test: *n* = 7, *p* < 0.03. ***G***, The decay time constant of sEPSCs at −60 mV (*p* = 0.171). ***H***, Time course of average RI (*n* = 7). One-way repeated-measures ANOVA, *p* = 0.004; *post hoc*: Bonferroni *t* test: 0–5 vs 10–15 min, *p* < 0.05; 0–5 vs 15–20 min, *p* < 0.01; *n* = 7). ***I***, ***J***, Alexa Fluor 594 (100 µm) fluorescence intensity along stellate cell dendrites (5 dendrites, 4 cells, 4 animals) at each time point. ***I***, Plot of the average fluorescence intensity along dendrites from the soma at 0–5 min (open circles) and 15–20 min (red circles). Arrows show the locations where Alexa Fluor 594 fluorescence intensity decreased to the basal level. ***J***, Time course of the diffusion distance of Alexa Fluor 594 along dendrites. One-way repeated-measures ANOVA, *p* = 0.014; *post hoc*: Bonferroni *t* test: 0–5 vs 10–15 min, *p* < 0.05; 0–5 vs 15–20 min, *p* < 0.05; *n* = 5.

Spermine, which is known to block GluA2-lacking AMPAR currents at depolarized potentials, was included in the pipette solution to compensate for the washout of endogenous spermine (Kamboj et al., 1995) and to identify the AMPAR subtype (Liu and Zukin, 2007). Spontaneous EPSCs in stellate cells are mediated by AMPARs, and have an inwardly rectifying *I–V* relationship and thus a low rectification index (Liu and Cull-Candy, 2000). When we recorded synaptic currents with Alexa 594 in the patch pipette, the average amplitude of sEPSCs at −60 mV was −34.2 ± 3.3 pA (*n* = 9) during the initial 5 min of recording. The current amplitude at +40 mV was 10.3 ± 1.0 pA and was markedly reduced compared with EPSCs at negative potentials. Thus, synaptic currents had an inwardly rectifying *I–V* relationship with a rectification index of 0.47 ± 0.1 (*n* = 9; Fig. 1*A*,*B*), indicating that stellate cells have a high proportion of synaptic GluA2-lacking AMPARs.

However, the rectification index of sEPSCs increased significantly to 0.59 ± 0.1 (vs RI_0-5 min_, *P* = 0.03, *n* = 9^a^; Fig. 1*B*) during 15–20 min of recording when Alexa 594 had presumably diffused into the distal dendrites, suggesting that Alexa 594 altered the properties of EPSCs. AMPAR-mediated synaptic currents in stellate cells show a wide range of rectification index values (Liu and Cull-Candy, 2002). To test whether cytoplasmic Alexa 594 preferentially altered the rectification index of EPSCs in stellate cells that express GluA2-lacking receptors, we examined the relationship between the initial RI (determined within 5 min) and the change in RI following 15–20 min of recording (Fig. 1*C*). The stellate cells with a lower initial RI showed a greater increase in RI. Thus, changes in the rectification index of synaptic currents occurred predominantly in stellate cells that initially displayed an inwardly rectifying *I–V* relationship (RI < 0.5). In seven cells with a low initial RI (<0.5), Alexa 594 did not alter the amplitude of EPSCs at −60 mV (0–5 min: −36.8 ± 3.6 pA; 15–20 min: −31.3 ± 3.7 pA; *p* = 0.11; *n* = 7^c^; Fig. 1*E*). However, the amplitude of EPSCs at +40 mV increased from 9.2 ± 1.1 to 11 ± 1 pA (*p* = 0.03; *n* = 7^d^; Fig. 1*F*). Consequently, the RI of EPSCs in stellate cells changed from 0.38 ± 0.03 at the beginning of the recording to 0.55 ± 0.05 at 15–20 min (*p* = 0.01; *n* = 7^b^; Fig. 1*D*). The increase in the RI occurred gradually and reached a plateau after 15–20 min of recording (*p* = 0.01; *n* =7^e^; Fig. 1*H*). We also monitored the diffusion of Alexa 594 by determining the fluorescence intensity along dendrites over the same time window. Alexa 594 was located in the soma immediately after obtaining the whole-cell configuration, and the fluorescence intensity in dendrites gradually increased such that the distal dendrites became visible. After 15–20 min, Alexa 594 fluorescence could be detected at dendritic sites that were at least 50 µm from the cell soma (57.0 ± 5.1 µm; *N* =5 ^f^; Fig. 1*I*,*J*). The time course of Alexa 594 diffusion matches the time taken for the change in the rectification index of EPSCs and is consistent with the idea that the diffusion of Alexa 594 from the soma to distal dendrites contributes to the gradual increase in EPSC rectification index. The decay time constant of EPSCs did not change over the same time window (0–5 min: 1.2 ± 0.1 ms; 15–20 min: 1.3 ± 0.1 ms; *n* = 7^g^; *p* = 0.171; Fig. 1*G*).

**Figure 2.**
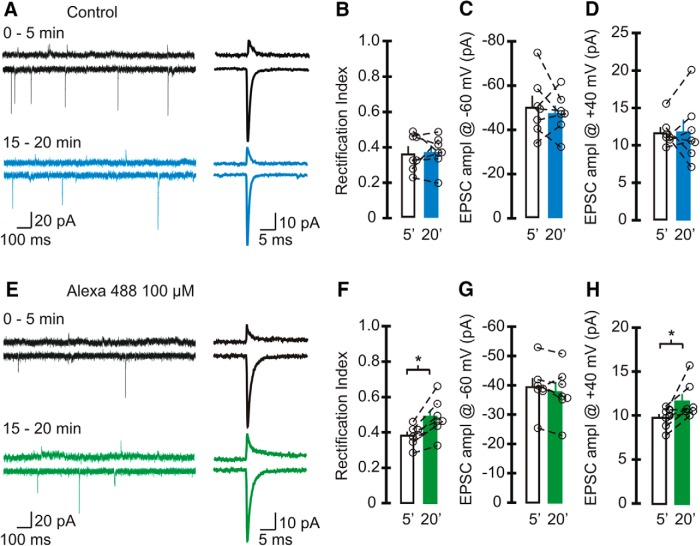
Alexa 488 increased the rectification index of sEPSCs in stellate cells. ***A–D***, Spontaneous EPSCs were recorded using a pipette solution that did not contain any Alexa Fluor dye (control). ***A***, Left, Example traces of sEPSCs recorded at +40 mV (top) and −60 mV (bottom) at 0–5 min (black trace) and after 15–20 min of recording (blue trace). Right, Average synaptic currents. ***B–D***, Average and individual RIs of sEPSCs (***B***) and amplitude at −60 mV (***C***), and +40 mV (***D***) in control cells with an initial RI < 0.5 (*n* = 7 cells, 6 animals). Neither the RI nor the amplitude of EPSCs changed within 20 min of recording. ***E–H***, Spontaneous EPSCs were recorded using a pipette solution that included 100 µm Alexa Fluor 488. ***E***, Left, Example traces of sEPSCs recorded with Alexa 488 in the recording electrode (black traces, 0–5 min; green traces, 15–20 min). Right, Average EPSCs. ***F***, RI increased after 15–20 min of recording (paired *t* test, *n* = 7 cells, 6 animals, *p* < 0.004). ***G***, EPSC amplitude at −60 mV did not change with time. ***H***, The amplitude of synaptic currents at +40 mV (paired *t* test, *p* = 0.025). Bar graphs represent the average, and open circles represent individual values.

As a control we also recorded EPSCs without including Alexa Fluor dye in the patch electrode (Fig. 2*A–D*). As expected, the EPSC amplitude at +40 mV (11.7 ± 0.7 pA) was markedly reduced compared with the current at −60 mV (−50.4 ± 5.1 pA), giving rise to an inwardly rectifying *I–V* relationship. The rectification index of EPSCs at 0–5 min (0.37 ± 0.04) was comparable to the initial RI observed with Alexa 594 (RI_0–5min_ = 0.38 ± 0.03; *p* = 0.8). After 20 min of recording, the RI of EPSCs did not change (RI_15–20min_ = 0.37 ± 0.04; *p* = 0.86; *n* = 7^h^), and the EPSC amplitude remained the same at both potentials^i, j^. Thus, the increase in RI was indeed due to the presence of Alexa 594. Note that the initial synaptic current amplitude at −60 mV in control cells was greater than the EPSC amplitude in the presence of Alexa 594 (0–5 min: −36.8 ± 3.6 pA; *p* = 0.05^t^; Table 2; Fig. 1*E* and 2*C*).

To determine whether the change in the RI of EPSCs is a common feature when Alexa Fluor dyes are included in the patch pipette, we also tested the effects of Alexa Fluor 488 (Fig. 2*E–H*). While the EPSC amplitude at −60 mV remained unaltered throughout the recording^l^, the current amplitude at +40 mV increased from 9.8 ± 0.4 pA at 0–5 min to 11.6 ± 0.8 pA at 15–20 min^m^. This led to an increase in the RI of EPSCs from 0.38 ± 0.02 to 0.48 ± 0.04 (*p* = 0.01; *n* = 7^k^). Therefore, Alexa Fluor dyes progressively increase the EPSC amplitude at positive potentials and render the AMPAR *I–V* relationship more linear in stellate cells. We observed a reduction in the EPSC amplitude at +40 mV compared with controls (0–5 min: Alexa 488, 9.8 ± 0.4 pA; control, 11.7 ± 0.7 pA; *p* = 0.05^u^; Table 2).

### Cytoplasmic Alexa Fluor 594 does not alter the amplitude of EPSCs mediated by GluA2-containing AMPARs

**Figure 3.**
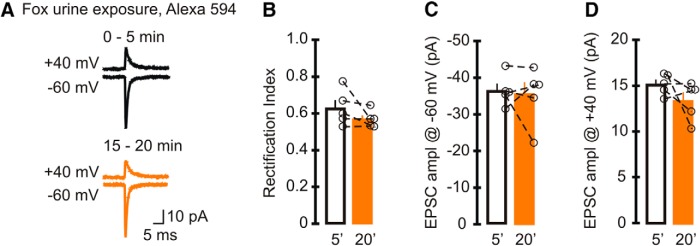
Intracellular Alexa Fluor 594 did not alter the rectification index of EPSCs mediated by GluR2-containing AMPA receptors. Mice were exposed to fox urine for 5 min, a treatment that increased GluA2-containing AMPARs and therefore the initial rectification index of EPSCs. Cerebellar slices were prepared 3 h later, and sEPSCs were recorded with a pipette solution that included Alexa 594. ***A***, Example trace of average synaptic currents (top, +40 mV; bottom, −60 mV; lines: black, 0–5 min; orange, 15–20 min). ***B–D***, Average and individual sEPSC rectification index (***B***) and amplitude (***C***) recorded at −60 mV and +40 mV (***D***). Neither the RI nor the amplitude of EPSCs changed after 15–20 min of recording (5 cells, 5 animals). Bar graphs represent the average, and open circles represent individual values.

Our results so far showed an increase in the RI of GluA2-lacking AMPAR-mediated currents when 100 µm Alexa 594 or 488 was included in the recording electrode. The magnitude of the increase in the RI of EPSCs was inversely proportional to the initial rectification (Fig. 1*C*), raising the possibility that synapses with high levels of GluA2-containing AMPARs were less sensitive to the effect of the dyes. To test this hypothesis, we increased the expression of GluA2 subunits in stellate cells by exposing mice to fox urine, an acute stressor that elevates synaptic GluA2-containing AMPARs, for 5 min (Liu et al., 2010). Three hours after exposure, we prepared cerebellar slices and recorded sEPSCs in stellate cells with 100 µm Alexa 594 in the pipette solution. As expected, the initial rectification index of EPSCs in stellate cells was high (0.63 ± 0.04; *n* = 5), was significantly larger than that in naive mice (*p* < 0.001), and was consistent with published results without Alexa 594 [RI = −1.5 × 18.2/(−41.2) = 0.67 ± 0.05; Liu et al., 2010; *p* = 0.421]. The average EPSC amplitude was 15.2 ± 0.5 pA at +40 mV and −36.5 ± 1.9 pA at −60 mV (Fig. 3*A–D*). In contrast to cells from naive mice, cytoplasmic Alexa 594 did not increase the RI (RI_15–20min_ = 0.57 ± 0.02; *p* = 0.2^n^) compared with the initial RI. EPSC amplitude at +40 mV and −60 mV remained unaltered at 15–20 min (amplitude at +40 mV for 15–20 min, 13.4 ± 0.9 pA, *p* = 0.2^p^; amplitude at −60 mV 15–20 min, −35.6 ± 3.2 pA, *p* = 0.78^o^; *n* = 5). Since Alexa 594 failed to alter the rectification index of EPSCs at GluA2-containing AMPAR synapses, our results support the idea that cytoplasmic Alexa Fluor dyes change the properties of synaptic currents mediated by GluA2-lacking AMPARs.

### A low concentration of Alexa Fluor 594 does not alter EPSC rectification

**Figure 4.**
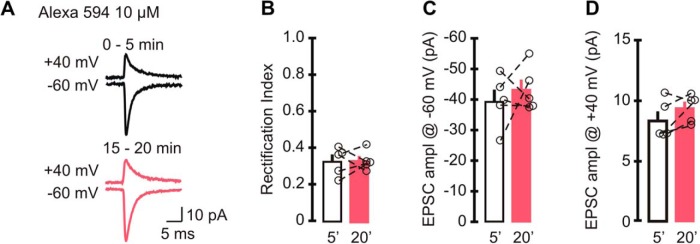
A low intracellular concentration of Alexa Fluor 594 did not alter the EPSC rectification index. ***A***, Example trace of average synaptic currents when 10 µm Alexa Fluor 594 was included in the recording electrode (top, +40 mV; bottom, −60 mV; lines: black, 0–5 min; pink, 15–20 min). ***B–D***, sEPSC rectification index (***B***) and amplitude (***C***) recorded at −60 mV and +40 mV (***D***). Neither the RI nor the amplitude of EPSCs changed at 15*–*20 min compared with initial values (5 cells, 4 animals). Bar graphs represent the average, and open circles represent individual values.

Since Alexa Fluor dyes are valuable tools that can be used to reveal the cytoarchitecture of live neurons, we tested whether Alexa 594 at a lower concentration still altered synaptic currents. We included 10 µm Alexa 594 in the patch electrode, and recorded sEPSCs at +40 and −60 mV, sequentially for 20 min (Fig. 4*A–D*). During the initial 5 min of recording, synaptic currents displayed an inwardly rectifying *I–V* relationship with a rectification index of 0.33 ± 0.03, which was indistinguishable from that of controls (without Alexa Fluor dyes, *p* = 0.144). In contrast to 100 µm Alexa 594, after 20 min the rectification index of EPSCs remained unaltered (RI_15–20 min_ = 0.33 ± 0.02 vs RI_0–5min_, *p* = 0.92^q^; *n* = 5), and the EPSC amplitude at +40 mV did not increase (amplitude at +40 mV for 0–5 min, 8.4 ± 0.7 pA; amplitude at +40 mV for 15–20 min, 9.4 ± 0.5 pA; *p* = 0.15^s^). The initial EPSC amplitude at −60 mV did not change throughout the recordings (amplitude at −60 mV for 0–5 min_,_ −39.6 ± 3.8 pA; amplitude at −60 mV for 15–20 min, −43.4 ± 3.3 pA; *p* = 0.5^r^). Therefore, although 100 µm Alexa Fluor dye can increase the rectification index of GluA2-lacking AMPAR-mediated currents, inclusion of a low concentration of Alexa 594 in patch electrode did not alter the rectification properties of synaptic currents in cerebellar stellate cells. However, we also noted a reduction in the EPSC amplitude at +40 mV compared with controls (0–5 min: 10 µm Alexa 594, 8.4 ± 0.7 pA; control, 11.7 ± 0.7 pA; *p* = 0.01^v^; Table 2), which is consistent with the decrease in the EPSC amplitude when Alexa 488 was used.

## Discussion

We have found that the inclusion of 100 µm Alexa Fluor 488 and 594 dyes in the pipette solution produced a gradual increase in the rectification index of synaptic currents mediated by GluA2-lacking AMPARs. This increase was associated with a rise in the amplitude of spontaneous EPSCs recorded at +40 mV, while the amplitude at −60 mV remained unchanged. In contrast, the rectification index of sEPSCs at synapses with GluA2-containing AMPARs was not sensitive to Alexa 594. This reveals that Alexa Fluor dyes can selectively alter the properties of synaptic currents conveyed by GluA2-lacking AMPARs at the parallel fiber–stellate cell synapse in the cerebellum. However, a lower concentration of 10 µm Alexa 594 did not alter the rectification index of EPSCs.

Can slow diffusion of small molecules, such as Alexa Fluor dyes, from the soma to dendrites explain a progressive increase in the rectification index? We showed that the diffusion of Alexa 594 along the dendrites increased during the 0–15 min window after obtaining the whole-cell configuration (Fig. 1*J*). This is the same time period during which intracellular the Alexa Fluor dyes produced an increase in the rectification of EPSCs (Fig. 1*H*). The diffusion of other small molecules between the neuronal somata and dendrites also takes ∼15 min. A study by Kamboj et al. (1995) has shown that endogenous polyamines wash out slowly from neurons when using a spermine-free internal solution, and this results in a time-dependent increase in the rectification index. This phenomenon also occurs within 15 min after obtaining the whole-cell configuration (Kamboj et al., 1995). Therefore, the diffusion of Alexa Fluor dyes along dendrites can give rise to a gradual increase in the rectification index.

What are the possible mechanisms for the cytoplasmic effect of Alexa Fluor dyes on the properties of EPSCs? The rectification index of AMPAR-mediated EPSCs depends on the subunit composition of receptors (Verdoorn et al., 1991; Mansour et al., 2001; Liu and Zukin, 2007), their interactions with auxiliary proteins such as transmembrane AMPAR regulatory proteins (TARPs; Tomita et al., 2005; Soto et al., 2007; Coombs and Cull-Candy, 2009), and intracellular spermine (Kamboj et al., 1995; Koh et al., 1995). Thus, cytoplasmic Alexa Fluor dyes may alter any of these three parameters, leading to a gradual increase in the rectification index. First, could Alexa Fluor dyes increase the expression of synaptic GluA2-containing receptors? Because the EPSC amplitude at −60 mV was unaltered throughout the recordings, it is unlikely that the presence of Alexa Fluor dyes increased the number of synaptic receptors and altered the AMPAR single-channel conductance. Second, could Alexa Fluor dyes promote the association of TARPs with AMPARs? TARPs prolong the deactivation time of AMPARs as well as increase the channel conductance (Cho et al., 2007). However, both the amplitude and the decay time constant of EPSCs at −60 mV did not change, indicating that the interaction between AMPARs and TARPs was not altered. Third, could Alexa Fluor dyes perturb the concentration of polyamines, which specifically block GluA2-lacking AMPARs at positive potentials? A possible explanation for the progressive increase in the RI of EPSCs is that cytoplasmic Alexa Fluor dyes reduce the ability of spermine to block GluA2-lacking AMPARs. Alternatively, Alexa Fluor dyes may deplete the available free spermine by buffering it or by blocking its access to the channel pore of GluA2-lacking AMPARs at positive potentials. This would lead to a rise in EPSC amplitude only at positive potentials, but not at −60 mV, resulting in an increase in the RI. Since polyamines in the recording pipette allow the determination of a synaptic AMPAR subtype (Koh et al., 1995), the inclusion of Alexa Fluor dyes could lead to errors in estimating the subtype of synaptic AMPARs. This raises the possibility that Alexa Fluor may also reduce the polyamine block of Ca^2+^-permeable kainate, nicotinic receptors, and inwardly rectifying potassium channels (Lopatin et al., 1994; Bowie and Mayer, 1995; Haghighi and Cooper, 1998). However, whether Alexa Fluor dyes alter the properties of other synaptic receptors and channels remains to be tested.

Are there other effects of Alexa Fluor dyes? Alexa Fluor dyes have been widely used during electrophysiological recordings in many different cell types (Mameli et al., 2007; Myoga et al., 2009; Zito et al., 2009; Higley and Sabatini, 2010; Maroteaux and Mameli, 2012) and have never been reported to be toxic within the time frame of the recording (Alford et al., 2009). However, in addition to changes in the EPSC rectification index, we also detected a reduction in the amplitude of sEPSCs immediately after obtaining the whole-cell configuration in the presence of Alexa Fluor dyes (Table 2). Because the RI was not altered at that time, the inhibitory effect was not subtype specific. This indicates that Alexa Fluor dyes should also reduce the EPSC amplitude at GluA2-containing synapses. Furthermore, we did not observe a gradual decrease in EPSC amplitude, suggesting that it is unlikely to be associated with the diffusion of Alexa Fluor dyes from the soma into the distal dendrites. Thus, one possible explanation is that Alexa Fluor dyes blocked AMPARs extracellularly as the dye escaped from the pipette due to the positive pressure that is applied while approaching the cell prior to seal formation. Our results suggest that Alexa Fluor dyes have unwanted effects on AMPAR-mediated synaptic transmission.

In conclusion, this study reveals that a widely used tool in live cell imaging can affect synaptic transmission in neurons. We show that both Alexa Fluor 488 and 594 alter the properties of AMPAR-mediated EPSCs at the parallel fiber–cerebellar stellate cell synapse. When present in the cytoplasm, Alexa Fluor dyes at 100 µm affect synaptic currents that are mediated via GluA2-lacking receptors, altering their rectification index, whereas lowering the Alexa 594 concentration to 10 µm did not produce this effect. Therefore, high concentrations of Alexa Fluor are not suitable for experiments that are designed to determine the subunit composition of synaptic currents. Because GluA3 and 4 AMPAR subunits are the most abundant in cerebellar stellate cells (Martin et al., 1993; Ripellino et al., 1998; Schwenk et al., 2014), whether Alexa Fluor dyes affect AMPARs composed of GluA1, such as those in hippocampal neurons (Wenthold et al., 1996; Zhu et al., 2000; Lu et al., 2009; Schwenk et al., 2014), remains to be tested. When Alexa 488 or 594 is included in pipette solution, it is, therefore, important to take into consideration the possibility that both dyes may alter the properties of synaptic currents, as we have shown here in cerebellar stellate cells.

## Synthesis

The decision was a result of the Reviewing Editor Vivian Budnik and the peer reviewers coming together and discussing their recommendations until a consensus was reached. A fact-based synthesis statement explaining their decision and outlining what is needed to prepare a revision is listed below. The following reviewer(s) agreed to reveal their identity: Stuart Cull-Candy

Both reviewers concur that the manuscript presents data that would be very useful for the field, particularly for those using Alexa dyes during in vivo studies. However, reviewers also had substantial comments that would need to be addressed experimentally before the manuscript can be published in eNeuro after re-review.

Addressing the following criticisms would be required before resubmission:

1. The pooling of data from another previous publication is unethical in a number of ways. Authors should conduct the experiments with the proper number of repetitions anew.

2. The authors should determine the diffusion of Alexa dyes from the soma to dendrites to assess whether the delayed effects can be explained by this diffusion.

3. The specifics of the Alexa dyes used (catalog#, form, stock solution composition) should be stated in the methods or results.

4. A dose-response should be conducted to determine if there is a concentration of Alexa dyes at which no deleterious effects are observed

6. Additional controls should be performed:

a. measure AMPAR-mediated EPSC rectification in neurons patched using spermine-free internal solution with or without Alexa dyes.

b. report the frequency of EPSC at 0-5' and 15-20' to take into account potential misrepresentation of synapses containing Ca-permeable vs Ca-impermeable AMPARs.

c. what is the specificity of the effect (i.e., do Alexa dyes affect other neuronal properties or cell viability)?

6. The authors should test the hypothesis that the effect of the dyes is extracellular by bath application of the dyes in neurons patched with an Alexa-free intracellular solution.

Reviewers had additional suggestions that the author may consider to include if available or obtainable within 2 months.

Reviewer 1:

Many electrophysiology studies use Alexa dyes to label neurons that they are recording from. Knowing how these dyes affect the intrinsic properties of the neurons is critical to interpretations of the data. This finding that Alexa dyes affect AMPA-mediated synaptic transmission is significant to the field and an important resource.There are a few points that should be addressed by the authors prior to publication (see note to authors).

The manuscript entitled "Alteration of AMPA receptor-mediated synaptic transmission by Alexa Fluor 488 and 594 in cerebellar stellate cells" describes a phenomenon by which AMPA-mediated (particularly GluA2-lacking AMPARs) synaptic transmission is altered using Alexa dyes, common reagents for dye-filling cells for electrophysiology experiments. The study offers an important finding that is broadly applicable to the field and should be a good resource going forward. There are a few points that should be addressed prior to publication:

1.The authors mention that the 15-20 min delay in alterations in the inwardly rectifying I-V relationship due to Alexa dyes is likely due to time to diffuse from soma to distal dendrites. Although correlative at this point, the authors could easily show this diffusion by imaging the cells over time and this would offer another dimension to the paper.

2.As a control, the authors should demonstrate that there is another dye that does not affect this AMPA-mediated transmission. Going forward, this will be important to the field. Conversely, it would be important to know if this is a more universal effect of all dye tracing.

3.The authors demonstrate that initial EPSC amplitude was affected at negative and positive membrane potentials; however, the effect was small at +40 mV and the authors pooled already published control data with new control data to get significance. Although it is understood that the data sets are comparable, this is not optimal. If more studies pooled previously published data with new data to raise their n and reach significance, it is likely that most everything would be significant at some point. However, I do believe that dye effects on initial EPSC amplitude, even if they are small and may be statistically insignificant, are still important.

4.The discussion focuses on the exact mechanism by which Alexa dyes confer deficits in AMPA-mediated synaptic transmission. At this point, knowing or postulating the exact mechanism seems less important compared to other issues that were not addressed. For example, another alternative mechanism is that the dye is simply toxic to the neurons, which is an important point. In addition, could Alexa dyes affect other receptor/transmitter systems? If the authors do not address this in the experiments, it should be discussed as a possibility and something to consider when designing these types of experiments in the future.

5.There are several sections of text where grammar/punctuation requires editing. This is particularly evident in the Abstract.

Reviewer 2

Many people use Alexa fluor dyes included in patch pipettes to allow them to visualize neurons. It is important to know whether this process alters the recorded currents. Here, the authors report that supplementing the intracellular solution with Alexa 488 and Alexa 594 does indeed alter synaptic AMPAR- currents mediated by calcium permeable AMPARs in cerebellar cells.

When performing whole-cell patch-clamp experiments, a variety of dyes have been included in patch pipettes (in different studies) to visualize neurons. It is clearly essential to know that such staining does not affect the recorded currents. Here, the authors report that supplementing the intracellular solution with Alexa fluor dyes (i.e. Alexa 488 and Alexa 594) alters AMPAR-mediated currents in cerebellar stellate cells. This is an unexpected result and, while no clear underlying mechanism is identified, this observation itself is a useful piece of information for the wider community studying excitatory central transmission.

That said, I feel the current manuscript could be improved in places:

1)I could find no catalogue number for Alexa 488 and 495 in the Methods or Results section. It is also important to state which forms of the Alexa molecules have been used (e.g. Hydrazide or Carboxylic Acid). It is essential to indicate the exact composition of the Alexa stock solution to justify the use of plain internal solution as control in figure 2A-D and throughout figure 4.

2)The authors explain the slow increase in RI they observed (plateauing at ∼15') by the slow diffusion of the Alexa molecules in the dendritic tree. Therefore, they need to show (using fluorescence imaging) how long it takes for the dye to entirely fill the stellate cell cytoplasm.

3)As an additional control, the authors should measure AMPAR-mediated EPSC rectification in neurons patched using spermine-free internal solution with or without Alexa dyes.

4)For the decreased EPSC amplitude observed in neurons patched with Alexa-containing intracellular solution (figure 4), the authors seem to favor the hypothesis of an extracellular effect on AMPAR. The authors should test this by bath applying Alexa while recording with an Alexa-free intracellular solution.

5)In these cells, the relative proportions of calcium-permeable (spermine-sensitive) and calcium-impermeable AMPARs is reported to vary from one synapse to another (according to Bats, Nat. Neurosc. 2012). This suggests synapses containing the largest proportion of calcium-permeable AMPARs might be expected to have sufficiently rectifying currents that they are undetectable at +40mV. Therefore, besides reporting the amplitude of averaged EPSCs at -60 and +40mV, the authors should probably also report frequency at 0-5' and 15-20' to take this into account.

## References

[B1] Abrahamsson T, Cathala L, Matsui K, Shigemoto R, DiGregorio DA (2012) Thin dendrites of cerebellar interneurons confer sublinear synaptic integration and a gradient of short-term plasticity. Neuron 73:1159–1172. 10.1016/j.neuron.2012.01.027 22445343

[B2] Albantakis L, Lohmann C (2009) A simple method for quantitative calcium imaging in unperturbed developing neurons. J Neurosci Methods 184:206–212. 10.1016/j.jneumeth.2009.08.004 19682493

[B3] Alford R, Simpson HM, Duberman J, Hill GC, Ogawa M, Regino C, Kobayashi H, Choyke PL (2009) Toxicity of organic fluorophores used in molecular imaging: literature review. Mol Imaging 8:341–354. 20003892

[B4] Bowie D, Mayer ML (1995) Inward rectification of both AMPA and kainate subtype glutamate receptors generated by polyamine-mediated ion channel block. Neuron 15:453–462. 7646897 10.1016/0896-6273(95)90049-7

[B5] Brenowitz SD, Regehr WG (2007) Reliability and heterogeneity of calcium signaling at single presynaptic boutons of cerebellar granule cells. J Neurosci 27:7888–7898. 10.1523/JNEUROSCI.1064-1007.2007 17652580 PMC6672738

[B6] Chadderton P, Schaefer AT, Williams SR, Margrie TW (2014) Sensory-evoked synaptic integration in cerebellar and cerebral cortical neurons. Nat Rev Neurosci 15:71–83. 10.1038/nrn3648 24434910

[B7] Cho C-H, St-Gelais F, Zhang W, Tomita S, Howe JR (2007) Two families of TARP isoforms that have distinct effects on the kinetic properties of AMPA receptors and synaptic currents. Neuron 55:890–904. 10.1016/j.neuron.2007.08.024 17880893

[B8] Coombs ID, Cull-Candy SG (2009) Transmembrane AMPA receptor regulatory proteins and AMPA receptor function in the cerebellum. Neuroscience 162:656–665. 10.1016/j.neuroscience.2009.01.004 19185052 PMC3217091

[B9] Ding JB, Takasaki KT, Sabatini BL (2009) Supraresolution imaging in brain slices using stimulated-emission depletion two-photon laser scanning microscopy. Neuron 63:429–437. 10.1016/j.neuron.2009.07.011 19709626 PMC2756148

[B10] Donevan SD, Rogawski MA (1995) Intracellular polyamines mediate inward rectification of Ca(2+)-permeable alpha-amino-3-hydroxy-5-methyl-4-isoxazolepropionic acid receptors. Proc Natl Acad Sci U S A 92:9298–9302. 7568121 10.1073/pnas.92.20.9298 PMC40972

[B11] Edelstein A, Amodaj N, Hoover K, Vale R, Stuurman N (2010) Computer control of microscopes using µManager. Curr Protoc Mol Biol Chapter 14:Unit14.20 10.1002/0471142727.mb1420s92 20890901 PMC3065365

[B12] Geiger JR, Melcher T, Koh DS, Sakmann B, Seeburg PH, Jonas P, Monyer H (1995) Relative abundance of subunit mRNAs determines gating and Ca2+ permeability of AMPA receptors in principal neurons and interneurons in rat CNS. Neuron 15:193–204. 7619522 10.1016/0896-6273(95)90076-4

[B13] Grienberger C, Chen X, Konnerth A (2015) Dendritic function in vivo. Trends Neurosci 38:45–54. 10.1016/j.tins.2014.11.002 25432423

[B14] Gu JG, Albuquerque C, Lee CJ, MacDermott AB (1996) Synaptic strengthening through activation of Ca2+-permeable AMPA receptors. Nature 381:793–796. 10.1038/381793a0 8657283

[B50] Haghighi AP, Cooper E (1998) Neuronal nicotinic acetylcholine receptors are blocked by intracellular spermine in a voltage-dependent manner. J Neurosci 18:4050–4062. http://www.jneurosci.org/content/18/11/4050.full 9592086 10.1523/JNEUROSCI.18-11-04050.1998 PMC6792788

[B15] Higley MJ, Sabatini BL (2010) Competitive regulation of synaptic Ca influx by D2 dopamine and A2A adenosine receptors. Nat Neurosci 13:958–966. 10.1038/nn.2592 20601948 PMC2910780

[B16] Huganir RL, Nicoll RA (2013) AMPARs and synaptic plasticity: the last 25 years. Neuron 80:704–717. 10.1016/j.neuron.2013.10.025 24183021 PMC4195488

[B17] Kamboj SK, Swanson GT, Cull-Candy SG (1995) Intracellular spermine confers rectification on rat calcium-permeable AMPA and kainate receptors. J Physiol 486:297–303. 10.1113/jphysiol.1995.sp020812 7473197 PMC1156521

[B18] Koh DS, Burnashev N, Jonas P (1995) Block of native Ca (2+)-permeable AMPA receptors in rat brain by intracellular polyamines generates double rectification. J Physiol 486:305–312. 10.1113/jphysiol.1995.sp020813 7473198 PMC1156522

[B19] Kullmann DM, Lamsa KP (2007) Long-term synaptic plasticity in hippocampal interneurons. Nat Rev Neurosci 8:687–699. 10.1038/nrn2207 17704811

[B20] Lamsa KP, Heeroma JH, Somogyi P, Rusakov DA, Kullmann DM (2007) Anti-Hebbian long-term potentiation in the hippocampal feedback inhibitory circuit. Science 315:1262–1266. 10.1126/science.1137450 17332410 PMC3369266

[B21] Liu SJ, Cull-Candy SG (2002) Activity-dependent change in AMPA receptor properties in cerebellar stellate cells. J Neurosci 22:3881–3889. 12019307 10.1523/JNEUROSCI.22-10-03881.2002 PMC6757644

[B22] Liu SJ, Zukin RS (2007) Ca2+-permeable AMPA receptors in synaptic plasticity and neuronal death. Trends Neurosci 30:126–134. 10.1016/j.tins.2007.01.006 17275103

[B23] Liu SQ, Cull-Candy SG (2000) Synaptic activity at calcium-permeable AMPA receptors induces a switch in receptor subtype. Nature 405:454–458. 10.1038/35013064 10839540

[B24] Liu Y, Formisano L, Savtchouk I, Takayasu Y, Szabó G, Zukin RS, Liu SJ (2010) A single fear-inducing stimulus induces a transcription-dependent switch in synaptic AMPAR phenotype. Nat Neurosci 13:223–231. 10.1038/nn.2474 20037575 PMC3140064

[B25] Lopatin AN, Makhina EN, Nichols CG (1994) Potassium channel block by cytoplasmic polyamines as the mechanism of intrinsic rectification. Nature 372:366–369. 10.1038/372366a0 7969496

[B26] Lu W, Shi Y, Jackson AC, Bjorgan K, During MJ, Sprengel R, Seeburg PH, Nicoll RA (2009) Subunit composition of synaptic AMPA receptors revealed by a single-cell genetic approach. Neuron 62:254–268. 10.1016/j.neuron.2009.02.027 19409270 PMC3632349

[B27] Magee JC (2000) Dendritic integration of excitatory synaptic input. Nat Rev Neurosci 1:181–190. 10.1038/35044552 11257906

[B28] Mameli M, Balland B, Luján R, Lüscher C (2007) Rapid synthesis and synaptic insertion of GluR2 for mGluR-LTD in the ventral tegmental area. Science 317:530–533. 10.1126/science.1142365 17656725

[B29] Mansour M, Nagarajan N, Nehring RB, Clements JD, Rosenmund C (2001) Heteromeric AMPA receptors assemble with a preferred subunit stoichiometry and spatial arrangement. Neuron 32:841–853. 11738030 10.1016/s0896-6273(01)00520-7

[B30] Maroteaux M, Mameli M (2012) Cocaine evokes projection-specific synaptic plasticity of lateral habenula neurons. J Neurosci 32:12641–12646. 10.1523/JNEUROSCI.2405-2412.2012 22956853 PMC6621263

[B31] Martin KC, Kosik KS (2002) Synaptic tagging–who’s it? Nat Rev Neurosci 3:813–820. 10.1038/nrn942 12360325

[B32] Martin LJ, Blackstone CD, Levey AI, Huganir RL, Price DL (1993) AMPA glutamate receptor subunits are differentially distributed in rat brain. Neuroscience 53:327–358. 8388083 10.1016/0306-4522(93)90199-p

[B33] Mathy A, Ho SSN, Davie JT, Duguid IC, Clark BA, Häusser M (2009) Encoding of oscillations by axonal bursts in inferior olive neurons. Neuron 62:388–399. 10.1016/j.neuron.2009.03.023 19447094 PMC2777250

[B34] Myoga MH, Beierlein M, Regehr WG (2009) Somatic spikes regulate dendritic signaling in small neurons in the absence of backpropagating action potentials. J Neurosci 29:7803–7814. 10.1523/JNEUROSCI.0030-3009.2009 19535592 PMC2840263

[B35] Panchuk-Voloshina N, Haugland RP, Bishop-Stewart J, Bhalgat MK, Millard PJ, Mao F, Leung W-Y, Haugland RP (1999) Alexa dyes, a series of new fluorescent dyes that yield exceptionally bright, photostable conjugates. J Histochem Cytochem 47:1179–1188. 10449539 10.1177/002215549904700910

[B36] Ripellino JA, Neve RL, Howe JR (1998) Expression and heteromeric interactions of non-N-methyl-D-aspartate glutamate receptor subunits in the developing and adult cerebellum. Neuroscience 82:485–497. 9466455 10.1016/s0306-4522(97)00296-0

[B37] Sabatini BL, Oertner TG, Svoboda K (2002) The life cycle of Ca(2+) ions in dendritic spines. Neuron 33:439–452. 11832230 10.1016/s0896-6273(02)00573-1

[B38] Schindelin J, Arganda-Carreras I, Frise E, Kaynig V, Longair M, Pietzsch T, Preibisch S, Rueden C, Saalfeld S, Schmid B, Tinevez J-Y, White DJ, Hartenstein V, Eliceiri K, Tomancak P, Cardona A (2012) Fiji: an open-source platform for biological-image analysis. Nat Methods 9:676–682. 10.1038/nmeth.2019 22743772 PMC3855844

[B39] Schwenk J, Baehrens D, Haupt A, Bildl W, Boudkkazi S, Roeper J, Fakler B, Schulte U (2014) Regional diversity and developmental dynamics of the AMPA-receptor proteome in the mammalian brain. Neuron 84:41–54. 10.1016/j.neuron.2014.08.044 25242221

[B40] Smith SL, Häusser M (2010) Parallel processing of visual space by neighboring neurons in mouse visual cortex. Nat Neurosci 13:1144–1149. 10.1038/nn.2620 20711183 PMC2999824

[B41] Soto D, Coombs ID, Kelly L, Farrant M, Cull-Candy SG (2007) Stargazin attenuates intracellular polyamine block of calcium-permeable AMPA receptors. Nat Neurosci 10:1260–1267. 10.1038/nn1966 17873873 PMC2430330

[B42] Swanson GT, Kamboj SK, Cull-Candy SG (1997) Single-channel properties of recombinant AMPA receptors depend on RNA editing, splice variation, and subunit composition. J Neurosci 17:58–69. 8987736 10.1523/JNEUROSCI.17-01-00058.1997 PMC6793687

[B43] Tomita S, Adesnik H, Sekiguchi M, Zhang W, Wada K, Howe JR, Nicoll RA, Bredt DS (2005) Stargazin modulates AMPA receptor gating and trafficking by distinct domains. Nature 435:1052–1058. 10.1038/nature03624 15858532

[B44] Verdoorn TA, Burnashev N, Monyer H, Seeburg PH, Sakmann B (1991) Structural determinants of ion flow through recombinant glutamate receptor channels. Science 252:1715–1718. 1710829 10.1126/science.1710829

[B45] Watt AJ, Cuntz H, Mori M, Nusser Z, Sjöström PJ, Häusser M (2009) Traveling waves in developing cerebellar cortex mediated by asymmetrical Purkinje cell connectivity. Nat Neurosci 12:463–473. 10.1038/nn.2285 19287389 PMC2912499

[B46] Wenthold RJ, Petralia RS, Blahos J II, Niedzielski AS (1996) Evidence for multiple AMPA receptor complexes in hippocampal CA1/CA2 neurons. J Neurosci 16:1982–1989. 8604042 10.1523/JNEUROSCI.16-06-01982.1996 PMC6578515

[B47] Wilms CD, Häusser M (2015) Reading out a spatiotemporal population code by imaging neighbouring parallel fibre axons in vivo. Nat Commun 6:6464 10.1038/ncomms7464 25751648 PMC4366501

[B48] Zhu JJ, Esteban JA, Hayashi Y, Malinow R (2000) Postnatal synaptic potentiation: delivery of GluR4-containing AMPA receptors by spontaneous activity. Nat Neurosci 3:1098–1106. 10.1038/80614 11036266

[B49] Zito K, Scheuss V, Knott G, Hill T, Svoboda K (2009) Rapid functional maturation of nascent dendritic spines. Neuron 61:247–258. 10.1016/j.neuron.2008.10.054 19186167 PMC2800307

